# Skin *Staphylococcus* species differentially modulate keratinocyte cytokine secretion in response to UVB

**DOI:** 10.1128/aem.01549-25

**Published:** 2026-04-14

**Authors:** Hannah J. Serrage, Mark D. Farrar, Andrew J. McBain, Joanne Pennock, Catherine O'Neill

**Affiliations:** 1 Centre for Dermatology Research, Division of Musculoskeletal and Dermatological Sciences, School of Biological Sciences, Faculty of Biology Medicine and Health, The University of Manchester 5292, Manchester, United Kingdom; 2 Division of Pharmacy and Optometry, School of Health Sciences, Faculty of Biology, Medicine and Health, The University of Manchester 5292, Manchester, United Kingdom; 3 Division of Infection, Immunity and Respiratory Medicine, School of Biological Sciences, Faculty of Biology Medicine and Health, The University of Manchester 5292, Manchester, United Kingdom; Washington University in St Louis, St. Louis, Missouri, USA

**Keywords:** ultraviolet radiation, sunlight, skin microbiome, bacteria, commensal

## Abstract

Skin acts as the body’s first line of defense against environmental insults including ultraviolet radiation (UVR) from sunlight and engages in a dynamic dialog with the resident skin microbiota, increasingly recognized for its role in shaping and educating the immune responses of the skin in both health and disease. However, how or indeed if the resident skin microbiota mediates inflammatory responses to sunlight remains unclear. To address this, we investigated the effects of five abundant members of the skin microbiota on cytokine secretion in human primary keratinocytes exposed to a single dose of UVB. Co-culture of primary keratinocytes with a defined five-species skin commensal community resulted in a broad increase in the secretion of innate immune mediators including interleukin-6 (IL-6), independent of UVB exposure. In the absence of UVB, *Staphylococcus epidermidis* was the dominant species, followed by *Staphylococcus hominis* within the five-species community. UVB induced a marked shift in community composition, characterized by increased proliferation of *S. hominis* and reduced *S. epidermidis* abundance, as confirmed by species-specific growth curve analyses. Assessment of species-specific effects using mono-associated host cells revealed *S. epidermidis* as the predominant contributor to the enhancement of immune mediator secretion. Without the presence of additional community members*,* UVB amplified *S. epidermidis*-induced cytokine secretion. However, co-culture of *S. epidermidis* with *S. hominis* attenuated the heightened inflammatory response to UVB typically associated with *S. epidermidis*, likely due to the reduced abundance of *S. epidermidis* following UVB exposure. These findings suggest that the resident skin microbiota may contribute to our inflammatory response to sunlight.

**IMPORTANCE:**

This study reveals that the skin microbiome may play a role in shaping inflammatory responses to UVB exposure. It provides evidence of organisms capable of both amplifying and mitigating inflammatory responses to UVB, highlighting the importance of microbial composition in photoprotection. These findings suggest individual responses to sunlight may be influenced not only by skin type but also by specific microbes present on the skin.

## INTRODUCTION

Human skin is home to a diverse and delicately balanced ecosystem comprising bacteria, fungi, viruses, mites, and archaea, collectively termed the skin microbiome. This microbial community contributes to host health through modulation of host immune responses, protection against invading pathogens, and metabolism of host products (1).

The skin is a multilayered structure, with the uppermost layer, the epidermis, being comprised primarily of keratinocytes. Keratinocytes have a key role in initiating responses to external stimuli including ultraviolet radiation (UVR) in sunlight. They function as skin “sentinels,” sensing and responding to damage- and pathogen-associated molecular patterns (DAMPs/PAMPs) through a range of pattern recognition receptors and effector molecules including cytokines and antimicrobial peptides. This results in the coordinated recruitment and interaction with neutrophils, dendritic cells, fibroblasts, and melanocytes that propagate inflammation (2, 3). Indeed, the critical role of keratinocytes in initiating and orchestrating responses to pathogens in diseases such as atopic dermatitis (4, 5) and psoriasis (6) has been highlighted.

UVR from sunlight comprises UVC (200–280 nm), UVB (280–315 nm), and UVA (315–400 nm). UVC and the majority of UVB is absorbed by Earth’s ozone layer; thus, ambient UVR reaching the Earth’s surface is approximately 95% UVA and 5% UVB. However, UVB has higher energy and is the waveband primarily responsible for acute exposure effects such as inflammation (sunburn), immunosuppression, and direct DNA damage, while chronic effects include photoaging and photocarcinogenesis (7–11). UVB-induced DNA damage, primarily cyclobutane pyrimidine dimers (CPDs), is thought to be the initiator of the sunburn response, an inflammatory cascade involving the recruitment of innate (primary) and adaptive (secondary) immune cells to the site of exposure (12, 13).

In recent years, studies have begun to unravel the complex dialog between the microbiome and sunlight in germ-free mice. Patra et al. showed that the presence of a microbiome could partially mitigate UVB-induced immunosuppression via enhanced secretion of innate immune mediators including interleukin-6 (IL-6) and IL-1β (14). However, these studies did not address the contributions of specific skin bacteria or account for the confounding change in gut bacteria in germ-free mice (15).

In the present study, we employed a microbial ecology approach with five highly prevalent and abundant members of the skin microbiome isolated from healthy volunteers: *Staphylococcus epidermidis, Staphylococcus hominis, Cutibacterium acnes, Corynebacterium tuberculostearicum,* and *Micrococcus luteus*. We explored how a model polymicrobial community (PMC) and specific bacterial species influence the production of cytokines by human primary keratinocytes and the modulation of this by UVR.

## MATERIALS AND METHODS

### Bacterial isolation and identification

The strains used in this study were isolated from healthy volunteers (study 2019-6208-10419, approved by the University of Manchester Ethics committee). Five participants between the ages of 23 and 32 (3 male and 2 female) were recruited for this study. All had Fitzpatrick skin types I–IV and had been delivered vaginally at birth (16). Exclusion criteria for this study are provided in supplementary materials. To collect microbiome samples, swabs were dipped into pre-warmed phosphate-buffered saline (PBS) and collected in duplicate into phosphate buffer (20 mM Na₂HPO₄, KH₂PO₄, 0.1% vol/vol Tween 80, and 0.03% wt/vol cysteine-hydrochloride, at pH 6.8; all obtained from Sigma-Aldrich) from the volar forearm, forehead, scalp, and toe web. Samples derived from each site were spread onto fastidious anaerobe agar + 5% vol/vol horse blood (Fisher Scientific), Wilkins-Chalgren anaerobe agar (WCA, Thermo Scientific), tryptic soy agar (TSA, Oxoid) ± 0.025% vol/vol Tween 80, MacConkey agar, mannitol salt agar, or Man-Rogosa-Sharpe agar incubated for up to 7 days under aerobic (37°C) or anaerobic conditions. Colonies of distinct morphology were subcultured onto WCA/TSA ± 0.1% vol/vol Tween 80, and once the purity was confirmed, they were frozen in brain-heart infusion + 0.5% wt/vol yeast extract and 30% vol/vol glycerol (Fisher Scientific, −80°C). Isolates were identified by 16S rRNA sequencing (Genomic Technologies Core Facility, University of Manchester) and presented as the strain genetically closest to the sample isolate below. Additional strain characteristics are provided in Table S1. Species with the highest relative abundance across skin sites were selected for inclusion within this study, according to Byrd et al. (1).

### Primary keratinocyte culture

Pooled (*n* = 3), normal human epidermal keratinocytes (NHEKs from juvenile foreskin, Promocell C-12005; passages 1–5) were routinely cultured in keratinocyte growth medium (KGM-2, Promocell). Keratinocytes were seeded into 12-well plates at 2–5 × 10⁴ cells/well and incubated (37°C, 5% vol/vol CO₂) until 80% confluency was reached. Cells were washed with PBS (Gibco) and either resuspended in PBS or inoculated with bacteria as described below.

### Bacterium-keratinocyte co-culture

*M. luteus, S. hominis,* and *S. epidermidis* were routinely cultured in tryptic soy broth (TSB, Oxoid) for 16 h and resuspended in 1 × 10^4^ CFU/mL in KGM-2. *C. tuberculostearicum* was cultured in TSB ± 0.1% vol/vol Tween 80 (Thermo Fisher) for 16 h and resuspended in 1 × 10^6^ CFU/mL as above. *C. acnes* was cultured in Wilkins-Chalgren anaerobe broth (WCB, Oxoid) for 48 h under anaerobic conditions (Don Whitley Scientific) and resuspended as described for *C. tuberculostearicum*. Following dilution to the appropriate concentration, strains were applied singly to NHEK monolayers (80% confluency) at 1 mL/well. NHEKs were co-cultured (37°C, 5% CO₂) for 90 (*C. acnes, C. tuberculostearicum,* and *M. luteus*), 60 (*S. hominis*), or 30 min (*S. epidermidis*) to achieve a microbial concentration of 1 × 10^4^ CFU/mL, prior to irradiation. To achieve equal proportions of each commensal member in the polymicrobial community prior to irradiation (1 × 10^4^ CFU/mL), strains were inoculated sequentially onto NHEK monolayers at concentrations described above with a cocktail of *M. luteus, C. tuberculostearicum,* and *C. acnes* (0.8 mL/well), followed by the addition of *S. hominis* (0.1 mL/well) and finally *S. epidermidis* (0.1 mL/well) at 30-min intervals. Following adherence, co-cultured NHEKs were washed twice with PBS prior to irradiation.

### Irradiation with UVB

A broadband UVB TL-12 lamp (Philips; 270–400 nm, peak 313 nm) was used with irradiance measured using a UVX radiometer (UV products, California) and UVX-31 detector. NHEK monolayers resuspended in PBS in the presence or absence of skin commensals were irradiated with UVB (16.5 and 33.0 mJ/cm^2^, erythemally weighted: 2.2 mJ/cm^2^ and 4.4 mJ/cm,^2^ respectively) with half the plate covered in UV-opaque material to act as a non-irradiated control. Following irradiation, PBS was replaced with KGM-2 and cultures incubated for 24 h (37°C, 5% vol/vol CO₂).

### Assessment of microbial growth

*S. hominis* and *S. epidermidis* were normalized to 1 × 10^4^ CFU/mL in KGM-2, inoculated into 24-well plates, and incubated for 30–60 min as described above to facilitate adherence. Media was replaced with PBS and cultures irradiated at 33 mJ/cm^2^ as described above. PBS was replaced with KGM-2 and microbial growth dynamics with or without exposure to UVB assessed every hour for 48 h via acquisition of absorbance readings at 600 nm.

### Cell viability and bacterial adherence

Twenty-four hours post-irradiation, cells were washed 3× with PBS and then trypsin (0.04% wt/vol). Ethylenediaminetetraacetic acid (EDTA, 0.03% wt/vol, Promocell) was applied for 7 min (37°C, 5% vol/vol CO₂). An equal volume of trypsin neutralizing solution was then applied to cells, and a sample was mixed with an equal volume of trypan blue (Gibco). Cell viability was determined using a hemocytometer (improved Neubauer). Alternatively, cell suspensions were collected for DNA extraction or bacterial viable counts as described above.

### Polymicrobial community composition assessment

Real-time qPCR analyses were performed to determine changes in the polymicrobial community composition in 24 h post-UVB exposure. Genomic DNA was extracted using the Masterpure Gram-positive DNA purification kit (Lucigen) and quality verified using a NanoDrop (260/280: 1.8-2.2; ThermoFisher Scientific, US).

Species-specific primers (Table 1) and species-specific standard curves (10^10^–10^2^ CFU/mL) were used to enable absolute quantification. For each organism comprising the polymicrobial community, genomic DNA was extracted from cultures at known CFU concentrations, and qPCR cycle threshold (Ct) values were correlated with CFU counts to generate standard curves.

**TABLE 1:** Primers, sequences, and standard curve equations used to quantify polymicrobial community composition

Species	Gene	Forward (5′–3′)	Reverse (5′–3′)	Interpolation^*a*^	Citation
*Corynebacterium tuberculostearicum*	recA	CGTAAGCTTCATCGACTGCACGAAGCACGC	CTCAAGCTTGGTTGATGAAGATGTG	y=−1.653ln(×) + 39.304	(17)
*Cutibacterium acnes*	recA	AGCTCGGTGGGGTTCTCTCATC	GCTTCCTCATACCACTGGTCATC	y=−0.987ln(×) + 48.424	(18)
*Micrococcus luteus*	recA	TGGGCATCTCCGTGGTSAA	TCTTGGTCTGGCCCTCGAACT	y=−1.697ln(×) + 44.014	(19)
*Staphylococcus epidermidis*	GyrA	ATGCGTGAATCATTCTTAGACTATGC	GAGCCAAAGTTACCTTGACC	y=−1.549ln(×) + 52.534	(20)
*Staphylococcus hominis*	GyrB	CCGATTTTATTATTAGATGATGTC	CTCTAATACGTTTTTGAAGTGTTT	y=−0.908ln(×) + 45.428	(21)

^*a*^ In each equation, y represents the Ct value and x represents CFU/mL.

Polymicrobial samples were run in parallel with each species-specific standard curve using the community Universal SYBR green Supermix kit according to the manufacturer’s instructions (Bio-Rad). Ct values from polymicrobial samples were interpolated against the corresponding standard curve to determine the genome equivalents (CFU/mL) for each species. These values were subsequently used to calculate the relative abundance of each organism within the community (Table 1). Total community counts were verified via CFU plating.

### Determination of cytokine concentrations

Pooled supernatant samples from NHEK treated with doses of UVB were collected 24 h post-exposure to UVB (10–20 mJ/cm²). The relative expression of 105 inflammatory cytokines from pooled supernatants was assessed using the Proteome Profiler Human XL Cytokine Array Kit (R&D Systems, Minneapolis, MN, USA), following the manufacturer’s instructions. NHEKs ± *M. luteus/S. hominis/S. epidermidis/C. tuberculostearicum/C. acnes* were exposed to UVB as above and assayed for tumor necrosis factor (TNF)-α, interleukin (IL)−8, IL-6, and CCL20 by ELISA according to the manufacturer’s protocol (Duoset, R and D systems, Biotechne). Data were normalized to keratinocyte cell number for analysis.

### Statistical analyses

All experiments were conducted at least three times with duplicate samples within each individual experiment. All data were normally distributed and analyzed by Student’s *t*-test or ANOVA with Tukey’s or Dunnett’s post hoc test as appropriate using Prism (GraphPad Software, California, US).

## RESULTS

### Exposure of NHEKs to a skin polymicrobial community broadly enhances cytokine secretion

**Fig 1:**
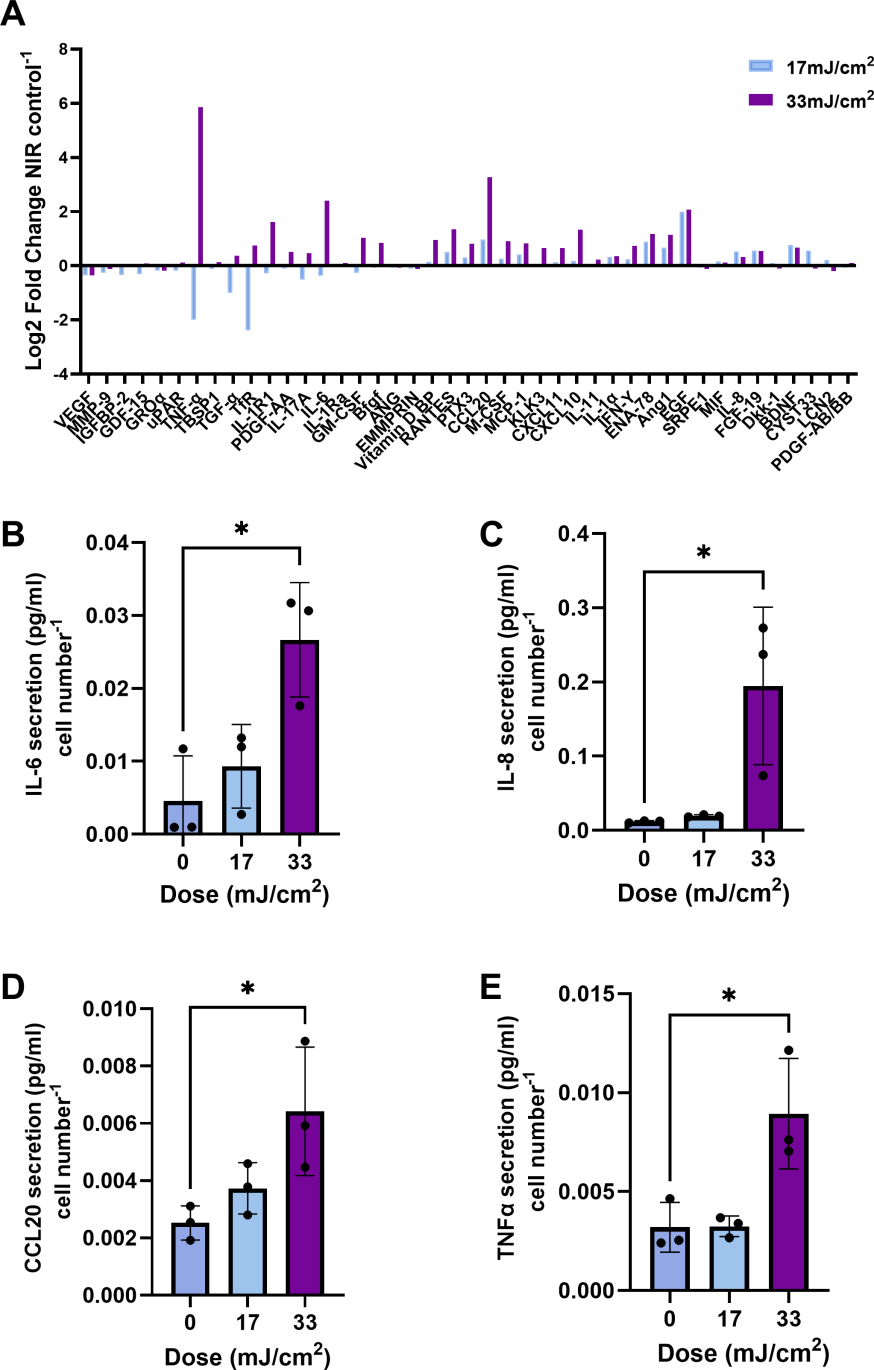
Dose-dependent effects on cytokine secretion. NHEKs (p3–5) cultured to 80% confluency were irradiated at 17–33 mJ/cm². Supernatants were collected 24 h post-irradiation and assessed via cytokine array (**A**) or via ELISA for IL-6 (**B**), IL-8 (**C**), CCL20 (**D**), and TNF-α (**E**). ELISA data are presented as mean ± SD. ****P < 0.0001, ***P < 0.001, **P < 0.01, and *P < 0.05, as determined via one-way ANOVA followed by Tukey test.

**Fig 2:**
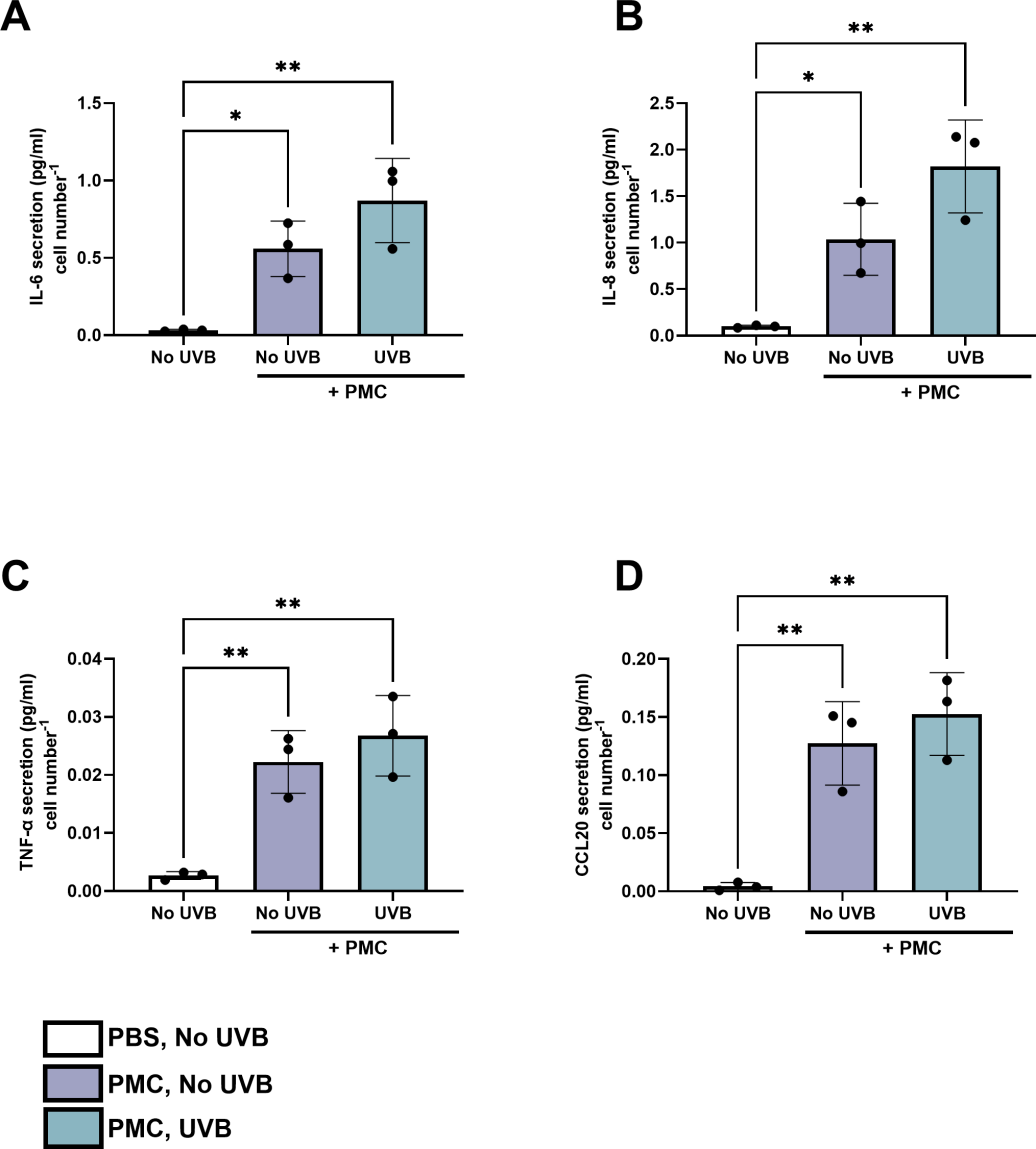
Exposure to a skin PMC elevates cytokine secretion regardless of UVB exposure. NHEKs (p2–5) were treated with PBS (PBS, no UVB) or *S. epidermidis, S. hominis, M. luteus, C. tuberculostearicum,* and *C. acnes* for 30–90 min and subsequently exposed to UVB (33 mJ/cm²). Supernatants were collected 24 h post-inoculation with each organism, and the effects of the skin microbial PMC ± UVB on IL-6 (**A**), IL-8 (**B**), TNF-α (**C**), and CCL20 (**D**) secretion were assessed using ELISA. **P < 0.01 and *P < 0.05, as determined via one-way ANOVA followed by Tukey test.

Dose-dependent cytokine responses to UVB were assessed using a cytokine array. Of the 105 cytokines surveyed, the expression of 43 was quantifiable (Table S2). Cytokines that exhibited either a >2-fold increase in expression following UVB exposure (IL-6, TNF-α, and CCL20) or high initial relative pixel density (IL-8) were selected for subsequent verification by ELISA (Fig. 1A; Table S2). Although UVB also induced a >2-fold increase in epidermal growth factor (EGF), this ligand plays a limited role in acute inflammatory signaling. Innate immune mediators showcasing clear dose-dependent effects were verified using ELISA to show that a dose of 33 mJ/cm^2^ but not 17 mJ/cm^2^ induced a significantly higher secretion of several cytokines, notably TNF-α (*P* < 0.05), IL-6 (*P* < 0.05), CCL20 (*P* < 0.05), and IL-8 (*P* < 0.05) compared to unirradiated cells (Fig. 1B through E). This dose-dependent increase in cytokine secretion was also accompanied by a 32% decrease in cell viability following exposure to a 33 mJ/cm^2^ dose of UVB (S1, *P* < 0.05). Exposure of NHEKs to a microbial community comprising *S. epidermidis, S. hominis, M. luteus, C. acnes,* and *C. tuberculostearicum* significantly enhanced the secretion of IL-6 (*P* < 0.05), IL-8 (*P* < 0.05), TNF-α, and CCL20 (*P* < 0.01; 33 mJ/cm^2^, Fig. 2). UVB did not significantly influence these cytokine responses. Community composition was then surveyed to decipher which members were contributing to observed cytokine responses.

### UVB alters the composition of a human skin polymicrobial community

**Fig 3:**
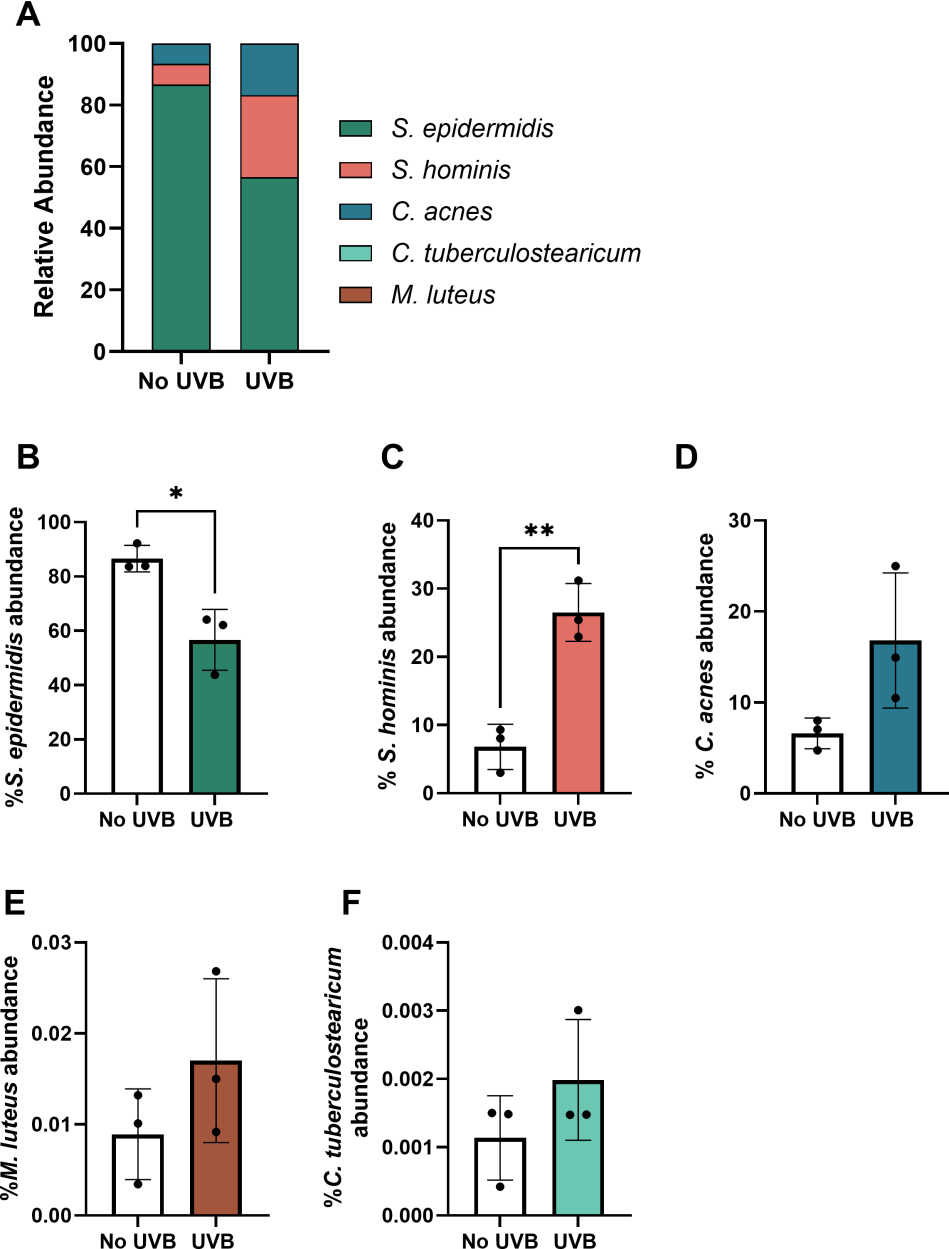
UVB impacts the relative abundance of skin commensals in a polymicrobial skin community. NHEKs (p2–5) were incubated with a polymicrobial community for 90 min, media replaced, and cultures exposed to 33 mJ/cm2 UVB or left unirradiated (no UVB). Polymicrobial community composition was assessed 24 h post-treatment via qPCR, where relative proportions of each organism were calculated via species-specific standard curves and presented as collated data (**A**, results for *C. tuberculostearicum* and *M. luteus* are not visible in the graph, as abundance was <1% of the total community) and UVB induced changes on individual species within the community of *S. epidermidis* (**B**), *S. hominis* (**C**), *C. acnes* (**D**), *M. luteus* (**E**), and *C. tuberculostearicum* (**F**). Data are presented as mean ± SD. **P < 0.01 and *P < 0.05, as determined via unpaired *t*-test.

**Fig 4:**
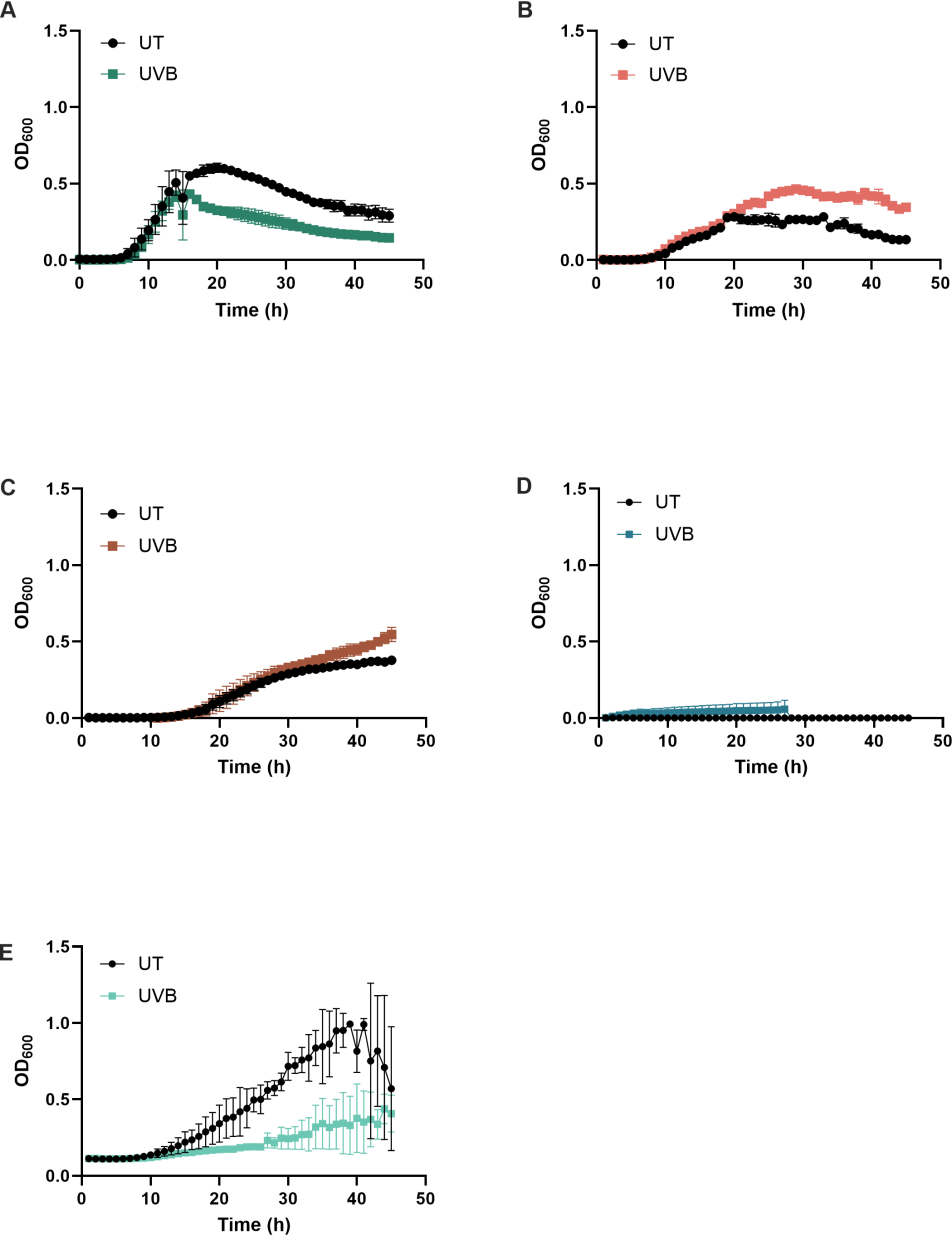
UVB differentially impacts skin commensal growth. *S. epidermidis* (**A**), *S. hominis* (**B**), *M. luteus* (**C**), *C. acnes* (**D**), and *C. tuberculostearicum* (**E**) were exposed to UVB 30–90 min post-inoculation (33 mJ/cm2), and effects on growth were assessed at 600 nm every 1 h for 48 h.

Community composition was validated via qPCR and total population counts confirmed using CFU counts (S2). Without exposure to UVB, the community comprised 86.6% *S. epidermidis,* 6.8% *S. hominis*, 6.6% *C. acnes,* and <1% *M*. *luteus* and *C. tuberculostearicum* (Fig. 3A). Exposure to UVB resulted in a shift in the relative abundance of colonizing species (Fig. 3A). Irradiation with UVB significantly elevated *S. hominis* abundance by 19.7% within the community compared to unirradiated controls (*P* < 0.01, Fig. 3C) but diminished the abundance of *S. epidermidis* by 30% relative to the unirradiated controls (*P* < 0.05, Fig. 3B). Other bacterial species were not significantly altered by UVB exposure 24 h post-exposure (Fig. 3D through F), but a mean increase in *C. acnes* was observed following exposure to UVB (Fig. 3D). To validate UVB-induced shifts in skin commensal abundance, growth curve assays were performed. UVB diminished *S. epidermidis* and *C. tuberculostearicum* exponential growth phase 19 h (Fig. 4A) and 25 h (Fig. 4E) post-irradiation, respectively, with limited recovery during the subsequent period of assessment (<48 h). In contrast, UVB extended the duration of the exponential growth phase for *S. hominis* at 22 h post-irradiation (Fig. 4B), and growth remained elevated relative to the untreated control for the remaining period of assessment (<48 h). UVB also slightly amplified the growth of *C. acnes* directly after irradiation (Fig. 4D) and growth of *M. luteus* 40 h post-irradiation (Fig. 4C). Despite slightly different conditions exploited to undertake growth curve assays (37°C growth curves vs. 37°C; 5% CO_2_ cell culture), growth curves corroborated with the relative abundance of each organism within the polymicrobial community, where growth of *M. luteus, C. acnes,* and *C. tuberculostearicum* remained limited 24 h post-irradiation (Fig. 4C through E). NHEKs were subsequently cultured with *S. epidermidis* or *S. hominis* to determine if microbial specific responses to UVB influenced keratinocyte cytokine secretion.

### UVB amplifies *S. epidermidis-*induced cytokine secretion

**Fig 5:**
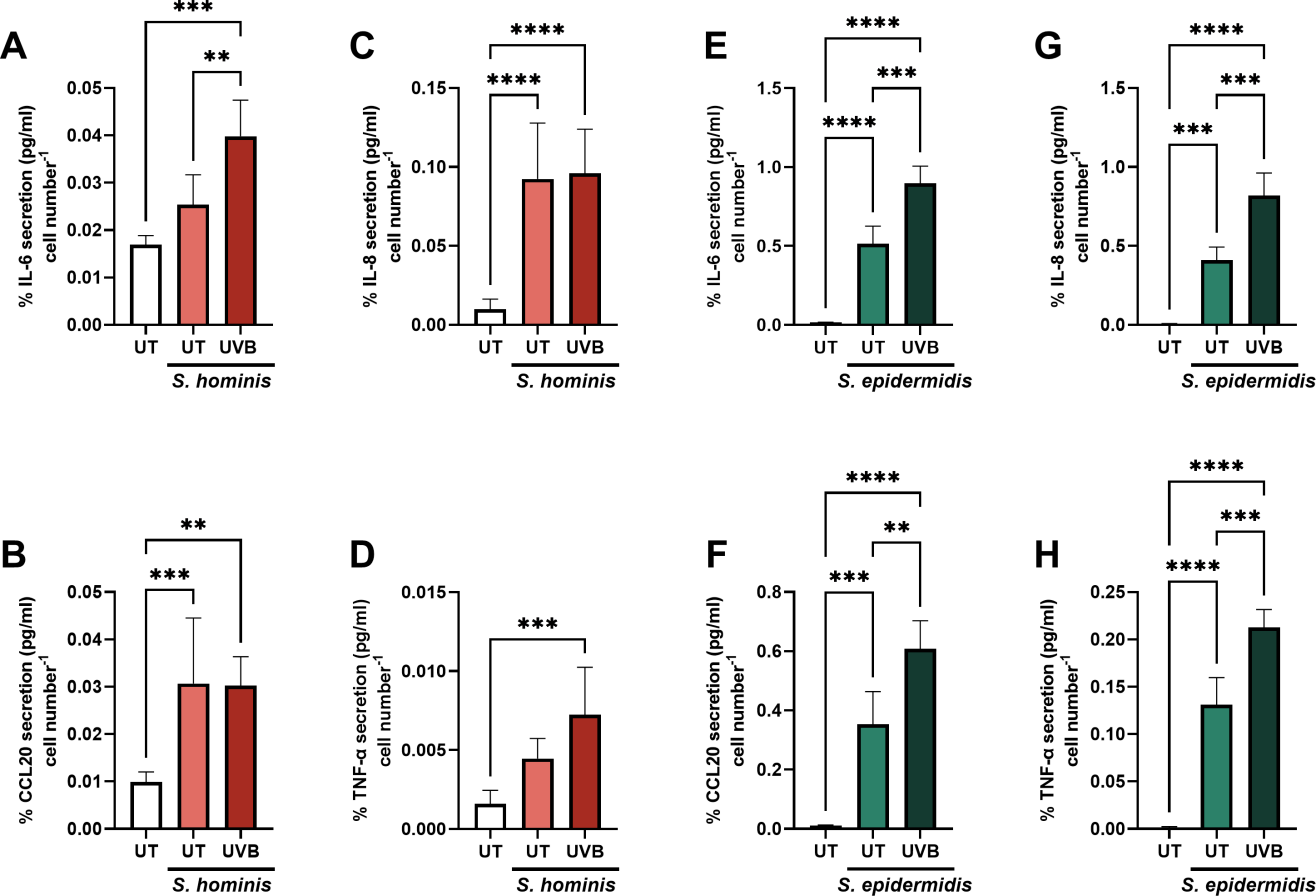
UVB exacerbates *S. epidermidis*-induced cytokine secretion, while *S. hominis* amplified secretion of IL-8 and CCL20 regardless of UVB exposure. NHEKs (p2–5) were incubated with media alone, *S. hominis,* or *S. epidermidis* for 30–60 min, media replaced with PBS, and exposed to UVB (0–33 mJ/cm²). Adherent bacteria were then co-cultured with NHEKs for 24 h, following which supernatants were collected to assess the effects on IL-6 (**A and E**), CCL20 (**B and F**), IL-8 (**C and G**), and TNF-α (**D and H**). Significance was assessed via one-way ANOVA followed by Tukey test, where **P < 0.01, ***P < 0.001, and ****P < 0.0001.

*S. hominis* and *S. epidermidis* accounted for >80% of the community (Fig. 3A), and thus it was hypothesized that these species were the primary contributors to the cytokine responses observed. To evaluate this, NHEKs were cultured with either *S. hominis* or *S. epidermidis* to achieve 1 × 10^4^ CFU/mL prior to irradiation. *S. hominis* enhanced the secretion of IL-8 (*P* < 0.0001) and CCL20 (*P* < 0.001) regardless of UVB exposure (Fig. 4B and C). UVB amplified the secretion of *S. hominis-*induced IL-6 relative to the bacterially treated control (*P* < 0.01, Fig. 4A). *S. epidermidis* elevated the secretion of IL-6 (*P* < 0.001), IL-8 (*P* < 0.001), CCL20 (*P* < 0.001), and TNF-α (*P* < 0.0001) regardless of UVB exposure (Fig. 5). Levels of cytokine induction irrespective of UVB exposure were more comparable in the polymicrobial and *S. epidermidis-*treated groups, relative to those exposed to *S. hominis* only (IL-8: PMC; 0.91 ± 0.30 pg/mL/cell, Fig. 1B, *S*. *hominis*: 0.09 ± 0.04 pg/mL/cell, and Fig. 4B *S*. *epidermidis*: 0.41 ± 0.08 pg/mL/cell, Fig. 5B). Exposure to UVB significantly amplified the secretion of *S. epidermidis-*induced IL-6, IL-8, TNF-α (*P* < 0.001), and CCL20 (*P* < 0.01; Fig. 5). Microbial survival was also surveyed 24 h post-irradiation, where, like in the polymicrobial community and growth curves, UVB enhanced *S. hominis* viability (S3a, *P* < 0.05) but diminished *S. epidermidis* viability (S3b, *P* < 0.05). In comparison, UVB exerted no significant effect on the viability of *M. luteus, C. tuberculosteraricum,* and *C. acnes* (S3C-E), supporting polymicrobial community analyses (Fig. 3). Taken together, these data indicate that *S. epidermidis* made a significant contribution to the nonspecific enhancement of cytokine secretion in the polymicrobial model due to its abundance and capacity to induce cytokine secretion in the absence of UVB.

*S. hominis* mitigates *S. epidermidis*-induced cytokine secretion following UVB exposure.

**Fig 6:**
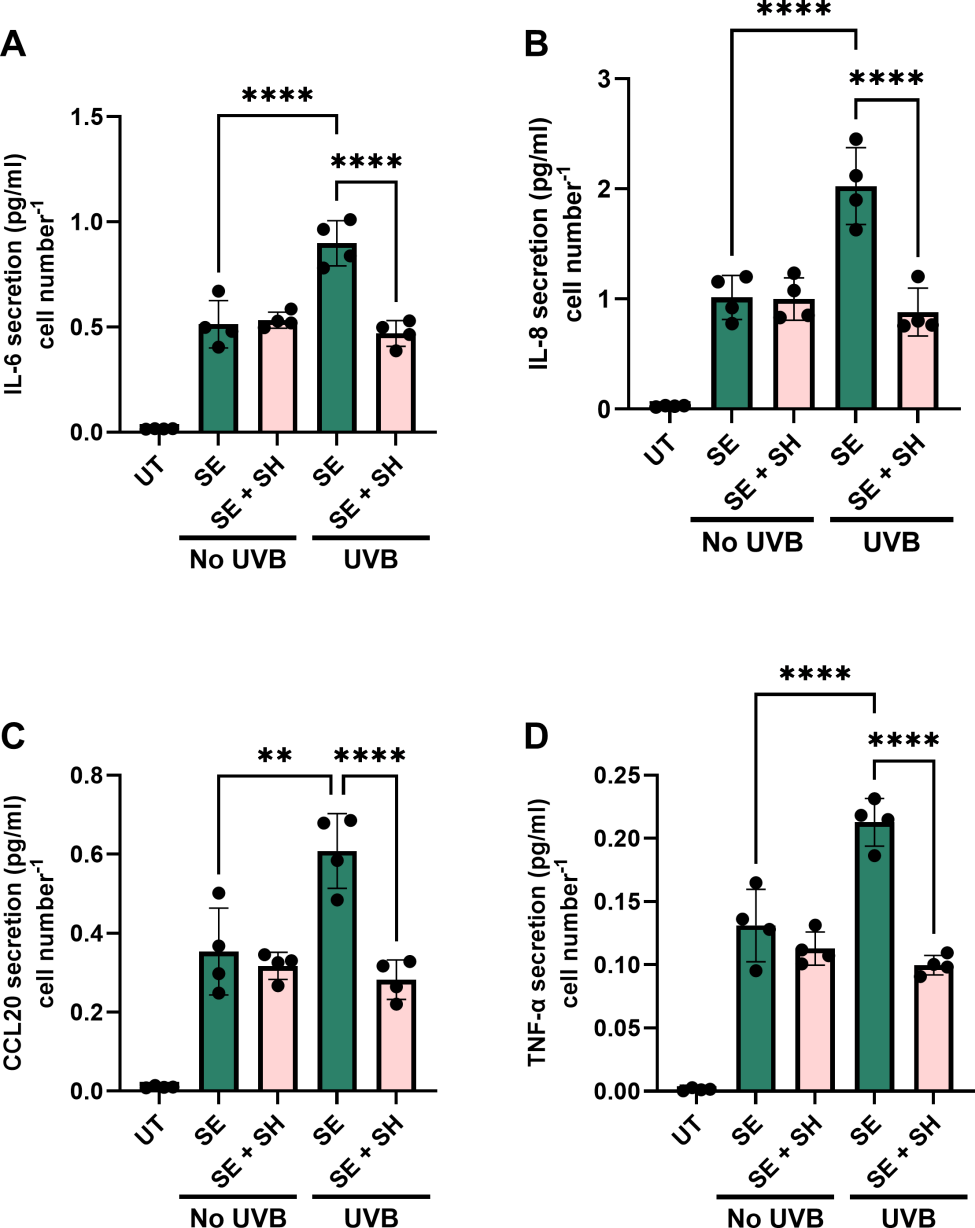
*S. hominis* diminishes *S. epidermidis-*enhanced cytokine responses to UVR. NHEKs (p2–5) were incubated with media, *S. hominis*, *S. epidermidis,* or both for 30–60 min, media replaced with PBS, and subsequently exposed to UVB (0–20 mJ/cm²). Twenty-hours post-microbial inoculation, supernatants were collected and effects on cytokine secretion assessed by ELISA (**A–D**). ****P < 0.0001, ***P < 0.001, **P < 0.01, and *P < 0.05, as determined via one-way ANOVA followed by Tukey test.

In the present study, UVB elevated *S. hominis* growth but diminished the growth of *S. epidermidis* (Fig. 2 and 3; Fig. S3). We hypothesized this shift in community composition could ameliorate UVB- amplified cytokine responses to *S. epidermidis*. In co-culture, *S. hominis* diminished *S. epidermidis-*exacerbated responses to UVB, reducing the secretion of IL-6 (Fig. 6A, *P* < 0.0001, 0.470 vs. 0.899 pg/mL/cell), IL-8 (Fig. 6B, *P* < 0.0001, 0.881 vs. 2.024 pg/mL/cell), CCL20 (Fig. 6C, *P* < 0.0001), and TNF-α (Fig. 6D, *P* < 0.0001) relative to the *S. epidermidis-*irradiated group.

## DISCUSSION

Humans and microbes alike have evolved under the influence of sunlight, and only recently have we begun to characterize the dynamic dialog between the skin and its resident microbiota in orchestrating responses to UVB. We have investigated this dialog, demonstrating the capacity of the abundant skin commensal *S. epidermidis* to amplify UVB-induced inflammatory responses, and in turn how shifts in community composition to enhance *S. hominis* abundance mitigate these responses.

When exploring individual bacterial contributions to the response of keratinocytes to UVB, in the present model, *S. epidermidis* proved the major constituent of the five species surveyed driving responses and inducing cytokine secretion regardless of UVB exposure. Unlike the collective response to the polymicrobial community, UVB enhanced *S. epidermidis-*induced IL-8 and IL-6 secretion. These are key players in the acute-phase response to sunlight exposure, triggering leukocytic infiltration into the skin and propagation of inflammation characteristics of erythema (22). The present study shows that *S. epidermidis* broadly enhanced secretion of all surveyed cytokines, which may contribute to dysregulated responses to sunlight. Recent studies have challenged the idea that *S. epidermidis* acts as a “harmless commensal” and instead operates as a “friend or foe” dependent upon the strain and context (23, 24). There is evidence that some strains of *S. epidermidis* dampen inflammatory responses to UVB (25, 26). However, the present study suggests that some strains are capable of exacerbating UVB-induced responses, at least in the context of this model. As this study exploited a single strain of *S. epidermidis,* future studies will endeavor to survey variability in the contribution of *S. epidermidis* to UVB-induced inflammatory responses. Exposure to UVB may act as an environmental stressor, resulting in the secretion of virulence factors such as phenol-soluble modulins and cysteine proteases including EcpA from *S. epidermidis* that perpetuate keratinocyte secretion of cytokines including IL-6, IL-8, CCL20, and TNF-α in response to UVB (27–29). The production of virulence factors is influenced by the microbial growth phase; for example, *S. epidermidis* expresses higher levels of a cell adhesion protein during the stationary phase compared to the exponential phase (30). In the present study, UVB exposure induced a premature transition of *S. epidermidis* into the stationary phase relative to the untreated control. This prolonged stationary phase may have resulted in the accumulation of virulence factors, potentially contributing to the heightened keratinocyte cytokine response observed following UVB treatment, despite a reduction in bacterial cell numbers 24 h post-irradiation.

*S. epidermidis* comprised a substantial portion of the polymicrobial community, yet keratinocyte responses to the mixed population differed considerably from those elicited by *S. epidermidis* or *S. hominis* alone. It was hypothesized that this was due to a UVB-induced shift in community composition, enhancing *S. hominis* and diminishing *S. epidermidis* abundance within the community, a trend validated by both qPCR and growth curves. The effects of UVB on skin microbial residents have been surveyed, revealing differential outcomes influenced by an array of factors including the presence or absence of chromophores. The excitation of these wavelength-dependent and species-specific light-absorbing molecules can influence microbial survival, resulting in either photoprotection or photodamage, the latter potentially causing microbial cell death (31–33). Absorbance profiles for bacterial strains used in this study were previously acquired to characterize chromophore expression across abundant skin commensals (34). Both *Staphylococcus* strains exhibited absorbance features suggestive of potential chromophore expression in the UVB spectrum. However, only *S. hominis* significantly reduced light transmission to a sensor, indicating notable UVB absorption. This suggests the presence of chromophores, such as flavins, the antioxidant properties of which may promote microbial survival, a role previously documented across various environmental bacterial species (35–38). *In vivo* studies also complement results observed in this study, indicating that exposure to UVB enhanced *S. hominis* abundance on the skin of patients with T-cell lymphoma (39).

Co-culture of *S. hominis* with *S. epidermidis* mitigated UVB-amplified cytokine responses induced by *S. epidermidis* alone. Single-species studies demonstrated that *S. hominis* elicited a comparatively weaker inflammatory response from NHEKs than *S. epidermidis*, suggesting that the increased abundance of *S. hominis* following UVB exposure may dampen *S. epidermidis*-driven cytokine response. This effect could stem from the differential effects of UVB on microbial growth, enhancing *S. hominis* proliferation while suppressing that of *S. epidermidis*. In addition, *S. hominis* secretes auto-inducing peptides with antagonistic properties against *S. epidermidis*, which may have also contributed to the modulatory effects observed in this study (40, 41).

The present study provides evidence for the role of *S. hominis* in mitigating *S. epidermidis-*exacerbated responses to UVB. However, other members of the initial polymicrobial community may also have contributed to these responses. Indeed, a mean increase in *C. acnes* and *M. luteus* was observed within the polymicrobial community following UVB exposure. Of note, *C. acnes* and *M. luteus* express the protein RoxP and the golden carotenoid pigment, sarcinaxanthin, respectively, that both exhibit antioxidant properties in mitigating oxidative stress induced by UVB or alternative sources and thus may have contributed to the responses observed (42–44).

The skin microbiome is increasingly being implicated in the mediation of inflammatory responses to sunlight and may also contribute to other processes such as melanogenesis (14, 45) and is also proving a key factor in the UVR-inducible condition, polymorphic light eruption (PLE) (46, 47). Removal of the skin microbiome from PLE patients using ethanol prior to irradiation reversed the cytokine imbalances commonly observed following exposure to UVR (48). Another recent study showcased a diminished overall abundance of *S. hominis* and *S. epidermidis* in patients with PLE, but with photo provocation resulting in a further decrease in *S. epidermidis* and a profound increase in the abundance of the carotenoid-expressing *Staphylococcus aureus,* whose chromophore expression may serve to promote survival and subsequent competitive colonization of the skin surface. Collectively, these data indicate the microbiota can influence an individual’s response to UVR in the context of disease, where an absence of *S. hominis* may predispose an individual to amplified responses to UVR (23, 32). The present data also show that certain bacterial species are capable of exacerbating responses to UVB. Collectively, these different observations suggest that an individual response to UVR may not be wholly related to the skin type but may also be associated with the species and possibly strains they harbor on their skin.

The present study used bacterial strains isolated from a range of anatomical sites. While these bacteria are prevalent across different skin sites, it is important to note that their relative abundance at each site may vary (49). Levels of photoexposure are also likely to differ between sites. In addition, growing evidence suggests both age and ethnicity contribute to skin microbiome composition (50, 51), with Streptococcus species predominating in young male children and *Cutibacterium* and *Staphylococcus* species in early adulthood (52). Our *in vitro* model comprising juvenile non-sun-exposed primary cells does not allow exploration of the influence of other epidermal cells such as melanocytes and Langerhans cells in UVB responses. There is crosstalk between these cell types, and evidence from *in vitro* 3D skin models suggests melanocytes may modulate antimicrobial peptide expression and bacterial colonization (53, 54). Further work will be required to develop physiologically relevant *ex vivo* models, including tissue biopsies, and polymicrobial communities that better reflect skin site, age, gender, and ethnicity.

In conclusion, this study suggests that community composition is critical in driving inflammatory responses to UVB, where the presence of certain species can alleviate strain-dependent amplification of these responses. Collectively, these data enhance the understanding of how UVB can manipulate the composition of microbial communities, how species interact to mediate responses to radiation, and inform on whether our resident microbiome might contribute to either photoprotection or harm.

## Data Availability

Data underlying the results presented in this publication are available at https://doi.org/10.48420/31444369. Strain information is provided in Table S1.

## SUPPLEMENTAL MATERIAL

Supplemental material aem.01549-25-s0001.pdf Tables S1 and S2; Fig. S1 to S3.

ASM does not own the copyrights to Supplemental Material that may be linked to, or accessed through, an article. The authors have granted ASM a non-exclusive, world-wide license to publish the Supplemental Material files. Please contact the corresponding author directly for reuse.
