# Supplementary material for: Skin Staphylococcus species differentially modulate keratinocyte cytokine secretion in response to UVB
Source: Appl Environ Microbiol. 2026 Apr 14;92(5):e01549-25. doi: 10.1128/aem.01549-25 (PMC13188905; doi:10.1128/aem.01549-25)
Supplement: Supplemental material — Tables S1 and S2; Fig. S1 to S3. [file aem.01549-25-s0001.pdf]

Supplementary Figures

Skin *Staphylococcus* species Differentially Modulate Keratinocyte Cytokine Secretion in Response to UVB

Hannah J. Serrage [a*], Mark D. Farrar [a], Andrew J. McBain [b], Joanne Pennock [c], Catherine O'Neill[a]

[a]Centre for Dermatology Research, Division of Musculoskeletal and Dermatological Sciences, School of Biological Sciences, Faculty of Biology Medicine and Health, The University of Manchester, Manchester, UK

[b]Division of Pharmacy and Optometry, School of Health Sciences, Faculty of Biology, Medicine and Health, The University of Manchester, Manchester, UK

[c]Division of Infection, Immunity and Respiratory Medicine, School of Biological Sciences, Faculty of Biology Medicine and Health, The University of Manchester, Manchester, UK

Key Words: Ultraviolet radiation, Sunlight, Skin Microbiome, Bacteria, Commensal
Running title: Staphylococcal Modulation of UVB Skin Response

Skin Microbiome Sampling Exclusion Criteria

All participants met the following inclusion criteria: were aged 18–65 years, had not used systemic antibiotics within the previous three months, had not consumed high-dose commercial probiotics (>10^8 CFU/ml), had not used topical antimicrobials within the past seven days, had not experienced a skin infection within the past six weeks, had washed their hair and body within the previous 24 hours, and were not diabetic.

Table 1: Strain and participant characteristics of organisms used in this study.

<table>
<tr><th>Species</th><th>Volunteer No*</th><th>Age</th><th>Ethnicity</th><th>Gender</th><th>Sample site</th></tr>
<tr><td>Staphylococcus epidermidis</td><td>4</td><td>23</td><td>White</td><td>Male</td><td>Forehead</td></tr>
<tr><td>Staphylococcus hominis</td><td>3</td><td>29</td><td>Arab</td><td>Male</td><td>Toe web</td></tr>
<tr><td>Micrococcus luteus</td><td>1</td><td>27</td><td>Asian</td><td>Female</td><td>Toe web</td></tr>
<tr><td>Cutibacterium acnes</td><td rowspan="2">2</td><td rowspan="2">32</td><td rowspan="2">Arab</td><td rowspan="2">Female</td><td>Forehead</td></tr>
<tr><td>Corynebacterium tuberculostearicum</td><td>Toe web</td></tr>
</table>

Table 2: Quantifiable Fold Change in Cytokine Expression Induced 24h post exposure to UVB at 17mJ/cm² or 33mJ/cm² relative to a Non-Irradiated Control.

	Fold changed relative to N-IR			Fold changed relative to N-IR	
**Marker**	**17mJ/cm²**	**33mJ/cm²**	**Marker**	**17mJ/cm²**	**33mJ/cm²**
**VEGF**	-0.35557	-0.3616	**CCL20**	**0.964893**	**3.266186**
**MMP-9**	-0.26994	-0.1107	**M-CSF**	0.254369	0.906035
**IGFBP-2**	-0.34493	-0.05432	**MCP-1**	0.423835	0.829941
**GDF-15**	-0.31845	-0.03097	**KLK3**	0.002129	0.655838
**GROα**	-0.19058	-0.18321	**CXCL11**	0.137796	0.65249
**uPAR**	-0.18345	0.114755	**CXCL10**	0.176935	1.333348
**TNF-α**	**-1.99971**	**5.852621**	**IL-11**	0.076948	0.227838
**TBSP1**	-0.12438	0.127146	**IL-1α**	0.325561	0.353849
**TGF-α**	-1.00557	0.368418	**IFN-Y**	0.241068	0.734395
**TfR**	-2.38279	0.737752	**ENA-78**	0.887442	1.176446
**IL-1R1**	-0.27689	1.622355	**Ang1**	0.674631	1.129646
**PDGF-AA**	-0.10602	0.515663	**EGF**	1.995046	2.076839
**IL-17A**	-0.50511	0.46701	**SRPE1**	0.052103	0.006357
**IL-6**	**-0.37211**	**2.401491**	**MIF**	0.158148	0.113326
**IL-1Ra**	-0.05685	0.096928	**IL-8**	**0.530523**	**0.322541**
**GM-CSF**	-0.25496	1.02407	**FGF-19**	0.556311	0.538723
**Bfgf**	-0.08103	0.844195	**Dkk-1**	0.1052	0.021629
**ANG**	-0.07324	0.046074	**BDNF**	0.76393	0.66869
**EMMPRIN**	-0.10761	0.005818	**CYST33**	0.558291	0.014217
**Vitamin D BP**	0.140698	0.947815	**LCN2**	0.201148	-0.19718
**RANTES**	0.502798	1.347816	**PDGF-AB/BB**	0.042703	-0.0284
**PTX3**	0.315541	0.817173			

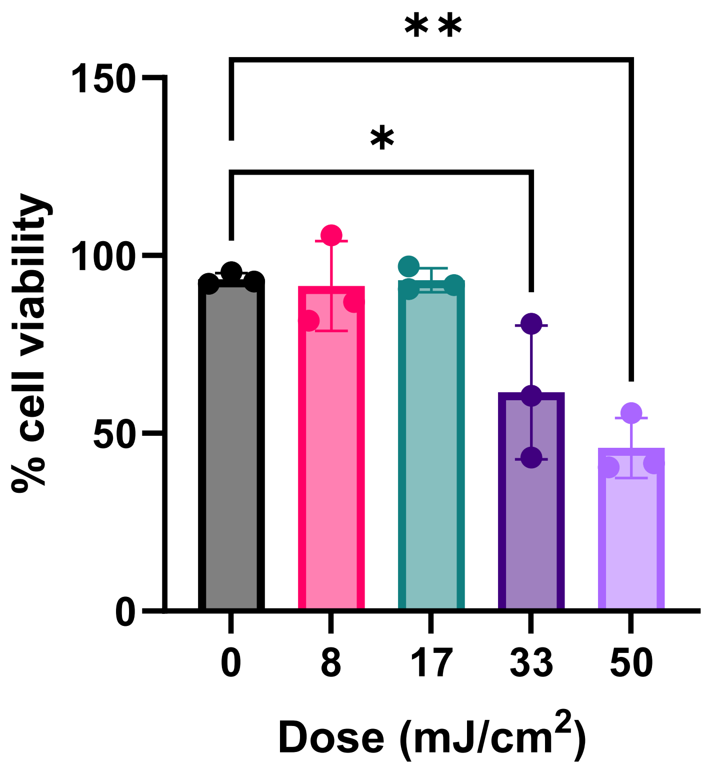

Figure 1: Dose Dependent Effects of UVB on Keratinocyte Viability. NHEKs (p3 – 5) cultured to 80% confluency were irradiated at 8 – 50mJ/cm² and viability assessed via trypan blue exclusion.

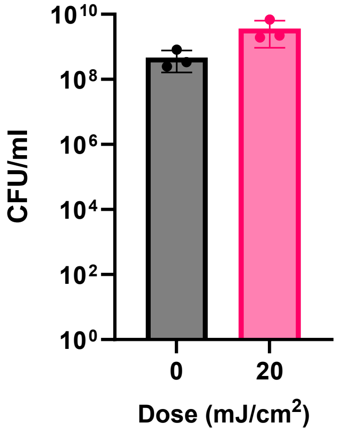

Figure 2: UVB exerts no significant effect on total microbial numbers. NHEKs (p2 – 5) treated with *S. epidermidis, S. hominis, M. luteus, C. tuberculostearicum* and *C. acnes* were exposed to UVB (0 – 20mJ/cm²) and relative changes in microbial number verified via plate counting. Data are presented as mean ± SD. **P<0.01, as determined via unpaired t-test.

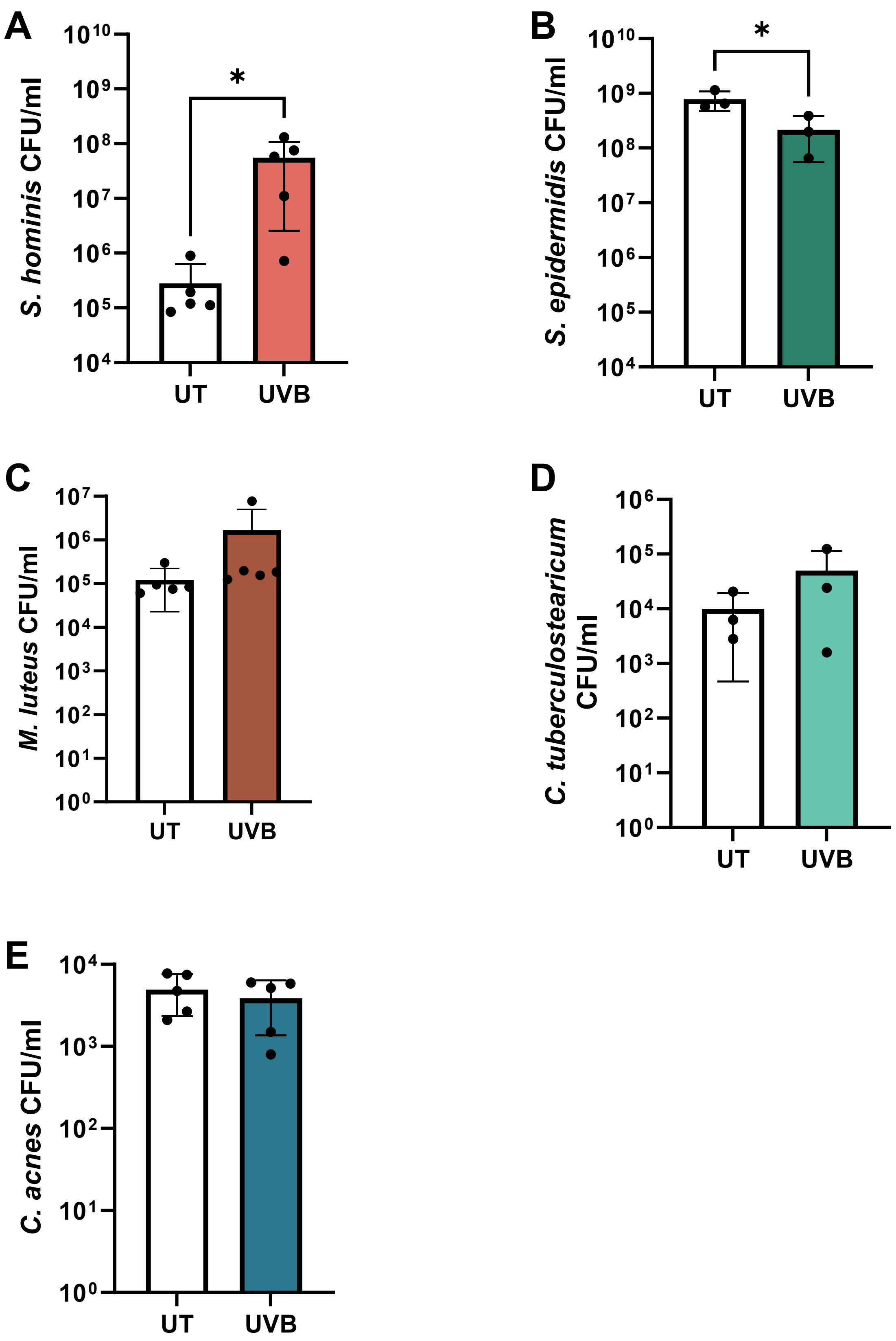

Figure 3: UVB mediates microbial survival in co-culture with NHEKs in a species-specific manner. NHEKs (p3 – 5) ± *S. hominis* (A) *S. epidermidis* (B), *M. luteus* (C), *C. tuberculoseaticum* (D) and *C. acnes* (E) were irradiated at 33/cm² and viability assessed via Miles and Misra 24h post irradiation. Significance assessed by t-test where **P<0.01 and *P<0.05.
